# Relationship between cigarette smoking and hearing loss in China: A cross-sectional study in Zhejiang province

**DOI:** 10.18332/tid/135440

**Published:** 2021-05-26

**Authors:** Dahui Wang, Yajun Zhu, Chenhui Li, Yi Wang, Shichang Wang, Shuang Wu, Shiyan Zhang, Liangwen Xu

**Affiliations:** 1 Department of Medicine, Hangzhou Normal University, Hangzhou City, China; 2 Department of Human Resources, Zhejiang Integrated Traditional and Western Medicine Hospital, Hangzhou City, China

**Keywords:** smoking, hearing loss, risk factor, general population

## Abstract

**INTRODUCTION:**

Hearing loss and cigarette smoking are major challenges that affect public health in China. Revealing the effect of smoking on hearing loss in the Chinese general population is critical for hearing health protection. We investigated the relationship between smoking status and hearing loss in China, especially in stratified sex and age groups.

**METHODS:**

A cross-sectional study was conducted on 4685 individuals aged 20– 80 years in Zhejiang province from 2016 to 2018, with audiometric testing for hearing loss and a structured questionnaire for collecting smoking status and covariates. Logistic regression was used to estimate the association between smoking and hearing loss.

**RESULTS:**

Cigarette smoking was not significantly associated with hearing loss in females and young males. In middle-aged males, after adjusting for covariates, current smokers and past smokers had a significantly higher prevalence of speech-frequency loss (OR=1.65; 95% CI: 1.17–2.33 and OR=1.88; 95% CI: 1.11–3.17; respectively) and high-frequency hearing loss (OR=2.01; 95% CI: 1.43–2.84 and OR=2.64; 95% CI: 1.50–4.66; respectively). In older males, only past smokers had a significantly higher prevalence of speech-frequency hearing loss than never smokers (OR=2.58; 95% CI: 1.38–4.85). Regarding middle-aged and older current smokers, a dose-dependent relationship between smoking intensity and hearing loss was found. Passive smoking was not significantly associated with an increased hearing loss risk in all the three male groups.

**CONCLUSIONS:**

The relationship between cigarette smoking and hearing loss varied according to gender and age. Therefore, the interventions for smoking need to be tailored according to age in males.

## INTRODUCTION

According to the World Health Organization (WHO), 466 million people globally suffer from disabling hearing loss, and more than 900 million people are expected to suffer from hearing loss by 2050. Hearing loss is a serious hazard to life quality and a burden to families and society, which costs the world economy $750 billion a year^1^. Many people with hearing loss receive inadequate treatment or rehabilitation^1-3^.

According to WHO, half of all cases of hearing loss can be prevented through public health measures such as following healthy ear care practices, reducing occupational and recreational noise exposure, screening of children for otitis media, avoiding ototoxic drug use, and providing hearing health education to the general population^4^. Therefore, identifying modifiable risk factors for hearing loss and implementing effective intervention measures to protect hearing health are essential^5^.

As reported by the 25-year Global Burden of Disease study (2017), hearing loss has become the second most common non-fatal problem affecting the life quality of Chinese population^6^. In China, more than 300 million people are smokers, and 740 million non-smokers exposed to passive smoking^7^. The results of previous studies have been inconsistent^8^, with some suggesting that cigarette smoking was associated with hearing loss^5,8-11^, whereas other studies reported no association^12,13^. Therefore, revealing the effect of smoking on hearing loss in the Chinese general population is critical for hearing health maintenance. In China, cigarette smoking was proven to be associated with speech-frequency hearing loss in occupational participants of the Dongfeng Motor Corporation in a cross-sectional study^8^. However, few studies have explored this relationship in the Chinese general population. According to the Summary of the 2015 China Adult Tobacco Survey Report delivered by Chinese Center for Disease Control and Prevention, the age-standardized smoking prevalence was 37.5% for males and 2.2% for females^14^, and in China, the distribution of smoking status varies among different age groups^10^. This is also likely to be the case for other potential factors that influence health. Additionally, aging is an independent risk factor for hearing loss^8^. Most previous studies mainly explored the relationship between smoking and hearing loss by adjusting for age, rather than analyzing the relationship in specific age subgroups after controlling for other covariates. Therefore, we hypothesized that the influence of cigarette smoking on hearing loss likely varies according to gender and age in China. In this study, we selected a large sample of the general population, collected smoking status and other factors previously reported to be associated with hearing loss, and measured nine hearing frequency bands. We investigated the relationship between smoking status and hearing loss in China, especially in stratified gender and age groups.

## METHODS

### Study participants

The study population was compiled using a multistage stratified cluster random sampling method in Zhejiang province from 2016 to 2018. Five cities (Hangzhou, Jiaxing, Huzhou, Quzhou, and Lishui) were randomly selected by stratifying the geographical distribution and city sizes based on the national nutrition and health survey in Zhejiang in 2012. One healthcare center was then randomly selected in each city, and all the participants in each healthcare center were selected by cluster sampling if they met the following inclusion criteria: 1) were local residents (who have lived there for more than 12 months), 2) were working in workplaces that do not meet the national noise pollution standards (exposure to non-occupational noise environment), and 3) underwent physical examination. The exclusion criteria were: 1) the presence of ear diseases like otitis media, and 2) aged <20 years or >80 years.

In total, 4930 persons who participated signed an informed consent form and completed a questionnaire and audiometry test in a hearing room. The survey results were obtained after excluding 245 participants with missing information on smoking-related problems or hearing data, those with ear problems such as otitis media, and those aged <20 years or <80 years. Finally, 4685 surveyed residents were analyzed, including 2386 males and 2299 females. To analyze the association between smoking status and hearing loss according to age, the population was divided into three age groups: young (20–39 years), middle-aged (40–59 years) and older group (60–80 years).

### Audiometry measures

Devices utilized in this research were the Madsen Itera clinical diagnostic audiometer and TDH39 headphones developed by GN Otometrics (Denmark). The audiometer was calibrated, and the audiometric tests were performed by trained technicians in a sound-proof chamber with noise levels below 30 dB. Pure-tone air-conduction hearing threshold testing at frequencies of 0.125 kHz, 0.25 kHz, 0.5 kHz, 1 kHz, 2 kHz, 3 kHz, 4 kHz, 6 kHz and 8 kHz was performed on both ears. To measure the reliability of participant responses, the 1 kHz frequency was tested twice in each ear. If the results differed by more than 10 dB, it was considered to be an unreliable response. When their answer was unreliable, we reconfirmed the procedure with them and reminded them to conduct the hearing test correctly. We computed the pure-tone average (PTA) at speech frequencies (0.5, 1, 2 and 4 kHz) and high frequencies (3, 4, 6 and 8 kHz). Hearing loss was defined as speech-PTA of ≥26 dB in the better ear, which is consistent with the WHO definition of clinically significant hearing loss and can identify patients with bilateral hearing loss and related functional impairments.

### Questionnaire

A pilot questionnaire survey was carried out before the custom-designed questionnaire was finally adopted. Prior to administering the questionnaire survey, investigators received professional training to ensure data quality. The questionnaire included items related to basic demographics including gender, age, education, and income level; information on diseases including hypertension, diabetes, hyperlipidemia, otitis media, migraine, and anemia; lifestyle and behavioral factors, including smoking, drinking, and earphone use; and workplace noise exposure. Education level was categorized as: elementary school or secondary school (<High School); High school; and junior college, undergraduate or postgraduate (>High School). Average monthly income was classified into three categories: low ≤4000; middle 4000–6000, and high ≥6000 RMB (1000 Chinese Renminbi about US$148, average 2017). Based on cigarette smoking history, participants were categorized as: self-reported never smokers (smoking less than one cigarette per day on average for less than one year, and exposure to passive smoke for less than 1 day per week); passive smokers (smoking less than one cigarette per day on average for less than 1 year and being exposure to passive smoking for more than one day per week); current smokers (smoking at least one cigarette per day for more than 1 year); and past smokers (stop smoking and keep non-smoking at least six months since cessation). Regarding drinking history, participants were categorized as: self-reported non-drinkers (less than one drink per week), past drinkers (abstinence for more than 6 months), and current drinkers (alcohol consumption at least once a week for 6 months or more).

For current smokers, daily cigarette consumption and years of smoking were also recorded. A smoking index was calculated by multiplying the number of cigarettes smoked per day by the number of years smoked. Current smokers were divided into two subgroups: light smokers (smoking index ≤150) and heavy smokers (smoking index >150), according to the two-digit grouping based on the median of smoking index.

### Statistical analysis

The study used Epidata V.3.1 (The Epidata Association, Odense, Denmark) for survey data entry, including error check and correction (double entry and validation). SPSS V.21.0 (SPSS for Windows, IBM Corp., Armonk, NY) was used to conduct all statistical analyses. Sample descriptive data were summarized using percentage distributions. Chi-squared analysis was used to compare differences between various covariates groups. Binary logistic regression was used to estimate the association between hearing loss and smoking status, after adjustment for other variables including age, gender, personal income, education level, alcohol consumption, frequency of headphone use, hypertension, diabetes, hyperlipidemia, and workplace noise. All reported p-values were twotailed, and p<0.05 was considered statistically significant.

## RESULTS

### Characteristics of the study population

The characteristics of hearing loss according to basic demographics, information on diseases, lifestyle and behavioral factors, and workplace noise exposure are presented in Table 1. Of the 4685 eligible participants, 1882 (40.2%) had speech-frequency hearing loss, and 2527 (53.9%) had high-frequency hearing loss. Gender, age, education status, personal income, cigarette smoking, alcohol consumption, workplace noise, frequency of use of headphones, hypertension, diabetes and hyperlipidemia were significantly associated with speech-frequency and high-frequency hearing loss (all p<0.001). Anemia and migraine were not significantly associated with both types of hearing loss (Table 1).

**Table 1:** Descriptive characteristics of survey participants (N=4685)

*Variables*	*Total*	*Speech-frequency hearing loss*			*High-frequency hearing loss*		
		*Yes*	*No*	p*	*Yes*	*No*	p*
	*n (%)*	*n (%)*	*n (%)*		*n (%)*	*n (%)*	
**Gender**							
Male	2386 (50.9)	1343 (56.3)	1043 (43.7)	<0.001	1004 (42.1)	1382 (57.9)	<0.001
Female	2299 (49.1)	1460 (63.5)	839 (36.5)		1154 (50.2)	1145 (49.8)	
**Age groups** (years)							
Young (20–39)	1393 (29.7)	1239 (88.9)	154 (11.1)	<0.001	1120 (80.4)	273 (19.6)	<0.001
Middle-aged (40–59)	2050 (43.8)	1249 (60.9)	801 (39.1)		892 (43.5)	1158 (56.5)	
Older (60–80)	1242 (26.5)	315 (25.4)	927 (74.6)		146 (11.8)	1096 (88.2)	
**Educational level**							
<High school	1787 (38.1)	877 (49.1)	910 (50.9)	<0.001	629 (35.2)	1158 (64.8)	<0.001
High school	1842 (39.3)	1217 (66.1)	625 (33.9)		961 (52.2)	881 (47.8)	
>High school	1056 (22.5)	709 (67.1)	347 (32.9)		568 (53.8)	488 (46.2)	
**Income** (RMB)							
<4000	1557 (33.2)	638 (41.0)	919 (59.0)	<0.001	391 (25.1)	1166 (74.9)	<0.001
4000–6000	1216 (26.0)	700 (57.6)	516 (42.4)		539 (44.3)	677 (55.7)	
≥6000	1912 (40.8)	1465 (76.6)	447 (23.4)		1228 (64.2)	684 (35.8)	
**Cigarette smoking**							
Never smoker	2181 (46.6)	1385 (63.5)	796 (36.5)	<0.001	1073 (49.2)	1108 (50.8)	<0.001
Passive smoker	1309 (27.9)	871 (66.5)	438 (33.5)		713 (54.5)	596 (45.5)	
Current smoker	948 (20.2)	469 (49.5)	479 (50.5)		326 (34.39)	622 (65.6)	
Past smoker	247 (5.3)	78 (31.6)	169 (68.4)		46 (18.6)	201 (81.4)	
**Alcohol drinking**							
Never drinker	3738 (79.8)	2356 (63.0)	1382 (37.0)	<0.001	1841 (49.3)	1897 (50.7)	<0.001
Former drinker	78 (1.7)	35 (44.9)	43 (55.1)		27 (34.6)	51 (65.4)	
Current drinker	869 (18.5)	412 (47.4)	457 (52.6)		290 (33.4)	579 (66.6)	
**Workplace noise** (non-occupational noise exposure)							
No or very little	3054 (65.2)	1879 (61.5)	1175 (38.5)	<0.001	1446 (47.3)	1608 (52.7)	<0.001
≥1/week	1102 (23.5)	665 (60.3)	437 (39.7)		511 (46.4)	591 (53.6)	
≥1/day	529 (11.3)	259 (49.0)	270 (51.0)		201 (38.0)	328 (62.0)	
**Frequency of use of headphones**							
Never use	3127 (66.7)	1634 (52.3)	1493 (47.7)	<0.001	1195 (38.2)	1932 (61.8)	<0.001
1/week	942 (20.1)	691 (73.4)	251 (26.6)		554 (58.8)	388 (41.2)	
1–2/day	530 (11.3)	412 (77.7)	118 (22.3)		351 (66.2)	179 (33.8)	
>3/day	86 (1.8)	66 (76.7)	20 (23.3)		58 (67.4)	28 (32.6)	
**Hypertension**							
No	3808 (81.3)	2547 (66.9)	1261 (33.1)	<0.001	2023 (53.1)	1785 (46.9)	<0.001
Yes	877 (18.7)	256 (29.2)	621 (70.8)		135 (15.4)	742 (84.6)	
**Diabetes**							
No	4505 (96.2)	2759 (61.2)	1746 (38.8)	<0.001	2139 (47.5)	2366 (52.5)	<0.001
Yes	180 (3.8)	44 (24.4)	136 (75.6)		19 (10.6)	161 (89.4)	
**Hyperlipidemia**							
No	4432 (94.6)	2713 (61.2)	1719 (38.8)	<0.001	2103 (47.5)	2329 (52.5)	<0.001
Yes	253 (5.4)	90 (35.6)	163 (64.4)		55 (21.7)	198 (78.3)	
**Anemia**							
No	4532 (96.7)	2704 (59.7)	1828 (40.3)	0.240	2082 (45.9)	2450 (54.1)	0.366
Yes	153 (3.3)	99 (64.7)	54 (35.3)		76 (49.7)	77 (50.3)	
**Migraine**							
No	4553 (97.2)	2731 (60.0)	1822 (40.0)	0.209	2109 (46.3)	2444 (53.7)	0.041
Yes	132 (2.8)	72 (54.5)	60 (45.5)		49 (37.1)	83 (62.9)	

* Statistical significance at p<0.05. RMB: 1000 Chinese Renminbi about US$148 (average 2017).

### Smoking status distribution in different genders and age groups

Among the 4685 participants analyzed, 50.9% (n=2386) were male, and 49.1% (n=2299) were female. Regarding age distribution, 29.7% (n=1393) were in the young group, 43.8% (n=2050) in middleaged group, and 26.5% (n=1242) in the older group. The chi-squared test indicated that the distribution of smoking status was statistically significantly different between male and female population. The proportions of current smokers and past smokers were 38.7% (n=923) and 9.5% (n=226) in males and 1.1% (n=25) and 0.9% (n=21) in female population, respectively. The distribution of smoking status was statistically significantly different among different age groups.

The middle-aged group had the highest proportion of current smokers (24.5%; n=502), whereas the highest proportion of passive smokers was in young group (36%; n=501). The proportion of past smokers increased with age, and the highest was 10.9% (n=135) in the older group (Table 2).

**Table 2:** Smoking status distribution in different gender and age subgroups (N=4685)

*Variables*	*Subgroups*	*Total n (%)*	*Smoking status*				p*
			*Never smoker n (%)*	*Passive smoker n (%)*	*Current smoker n (%)*	*Past smoker n (%)*	
**Gender**	Male	2386 (50.9)	724 (30.3)	513 (21.5)	92 (38.7)	226 (9.5)	<0.001
	Female	2299 (49.1)	1457 (63.4)	796 (34.6)	25 (1.1)	21 (0.9)	
**Age group** (years)	Young (20–39)	1393 (29.7)	672 (48.2)	501 (36.0)	200 (14.4)	20 (1.4)	<0.001
	Middle-aged (40–59)	2050 (43.8)	866 (42.2)	590 (28.8)	502 (24.5)	92 (4.5)	
	Older (60–80)	1242 (26.5)	643 (51.8)	218 (17.6)	246 (19.8)	135 (10.9)	

* Statistical significance at p<0.05

### Relationship of smoking status with hearing loss in males and females

Smoking status was significantly associated with speech-frequency and high-frequency hearing loss in males (p<0.001), but not in females (p>0.05). In males, after adjusting for other factors, current smoking and past smoking showed significantly increased risks for speech-frequency hearing loss compared with never smokers, with odds ratios (ORs) of 1.64 (95% CI: 1.27–2.11) and 2.16 (95% CI: 1.47–3.17), respectively (Table 3). Current smoking and past smoking were significantly associated with an increased risk of high-frequency hearing loss compared with never smokers, with ORs of 1.80 (95% CI: 1.38–2.34) and 2.38 (95% CI: 1.51–3.75), respectively (Table 3). Passive smokers did not have a significantly increased risk of both speech-frequency and high-frequency hearing loss in males compared to never smokers (Table 3)

**Table 3:** Adjusted odds ratio for prevalence of hearing loss with different smoking status in males and females (N=4685)

*Variables*	*Male (n=2386)*			*Female (n=2299)*		
	*AOR*	*95% CI*	p*	*AOR*	*95% CI*	p*
**Speech-frequency hearing loss**						
Never smokers (Ref.)	1		<0.001	1		0.213
Passive smokers	1.19	(0.89–1.59)	0.248	1.25	(0.99–1.57)	0.062
Current smokers	1.64	(1.27–2.11)	<0.001	1.77	(0.69–4.56)	0.237
Past smokers	2.16	(1.47–3.17)	<0.001	1.05	(0.35–3.18)	0.936
**High-frequency hearing loss**						
Never smokers (Ref.)	1		<0.001	1		0.990
Passive smokers	1.27	(0.95–1.70)	0.101	1.04	(0.83–1.29)	0.749
Current smokers	1.80	(1.38–2.34)	<0.001	0.99	(0.38–2.60)	0.990
Past smokers	2.38	(1.51–3.75)	<0.001	0.96	(0.31–2.98)	0.941

AOR: adjusted odds ratio; adjusted for the covariates of age, education level, income level, drinking, workplace noise, frequency of earphone use, hypertension, diabetes, and hyperlipidemia.

* Statistical significance at p<0.05. Ref.: reference group.

### Relationship between smoking status and hearing loss according to age in males

In the young group (20–39 years), smoking status was not significantly associated with speech-frequency and high-frequency hearing loss (p>0.05). In the middleaged group (40–59 years), cigarette smoking was significantly associated with both speech-frequency and high-frequency hearing loss (p<0.05). After adjusting for other variables, compared with never smokers, current smokers and past smokers had a significantly higher prevalence of speech-frequency hearing loss, with OR of 1.65 (95% CI: 1.17–2.33) and 1.88 (95% CI: 1.11–3.17), respectively. Current smokers and past smokers had a significantly higher prevalence of high-frequency hearing loss, with ORs of 2.01 (95% CI: 1.43–2.84) and 2.64 (95% CI: 1.50– 4.66), respectively. In the older group (60–80 years), compared with never smokers, only past smokers had a significantly higher prevalence of speech-frequency hearing loss, with OR of 2.58 (95% CI: 1.38–4.85). Smoking status was not significantly associated with high-frequency hearing loss (Table 4). We also analyzed the other covariates that have significant effect on hearing loss and the results are given in the Supplementary file Table S1. In young adults, besides smoking, drinking, workplace noise, hypertension and diabetes were significantly associated with speech-frequency hearing loss. Workplace noise and hypertension were significantly associated with high-frequency hearing loss. In the middle-aged group, besides smoking, workplace noise, hypertension and diabetes were significantly associated with speech-frequency hearing loss. Hypertension was significantly associated high-frequency hearing loss. In the older group, hypertension and hyperlipidemia were associated with speech-frequency hearing loss, and workplace noise with high-frequency hearing loss.

**Table 4:** Adjusted odds ratio for prevalence of hearing loss with different smoking status in three age subgroups in males (N=2386)

*Variables*	*Young (n=699) (20–39 years)*			*Middle-aged (n=1053) (40–59 years)*			*Older (n=634) (60–80 years)*		
	*AOR*	*95% CI*	p*	*AOR*	*95% CI*	p*	*AOR*	*95% CI*	p*
**Speech-frequency hearing loss**									
Never smokers (Ref.)	1		0.271	1		0.001	1		0.032
Passive smokers	1.42	(0.79–2.55)	0.237	0.93	(0.62–1.39)	0.728	1.40	(0.74–2.66)	0.306
Current smokers	1.61	(0.86–3.02)	0.136	1.65	(1.17–2.33)	0.004	1.29	(0.79–2.10)	0.309
Past smokers	3.27	(0.74–14.40)	0.117	1.88	(1.11–3.17)	0.019	2.58	(1.38–4.85)	0.003
**High-frequency hearing loss**									
Never smokers (Ref.)	1		0.246	1		<0.001	1		0.378
Passive smokers	1.09	(0.68–1.75)	0.726	1.25	(0.85–1.85)	0.255	1.36	(0.52–3.51)	0.529
Current smokers	1.55	(0.94–2.53)	0.083	2.01	(1.43–2.84)	<0.001	1.01	(0.49–2.08)	0.974
Past smokers	2.35	(0.56–9.79)	0.242	2.64	(1.50–4.66)	0.001	2.19	(0.84–5.71)	0.109

AOR: adjusted odds ratio; adjusted for the covariates of age, education level, income level, drinking, workplace noise, frequency of earphone use, hypertension, diabetes, and hyperlipidemia.

* Statistical significance at p<0.05. Ref.: reference group.

### Relationship between smoking intensity and the risk for hearing loss according to age in males

In the middle-aged group and older age group, the current smokers were further divided into two groups based on the smoking index, light smokers and heavy smokers. Compared with the light smokers, the heavy smokers had a higher risk for speech-frequency hearing loss in older group, with ORs of 2.35 (95% CI: 1.08–5.14), and also had a higher risk for high-frequency hearing loss in middle-aged group, with ORs of 1.89 (95% CI: 1.23–2.91, p<0.05) (Table 5).

**Table 5:** Adjusted odds ratio for prevalence of hearing loss with different smoking intensity in middle-aged group and older group of males

*Variables*	*Middle-aged (n=489) (40–59 years)*			*Older (n=237) (60–80 years)*		
	*AOR*	*95% CI*	p*	*AOR*	*95% CI*	p*
**Speech-frequency hearing loss**						
0<SI≤150 (Ref.)	1			1		
SI>150	1.36	(0.90–2.05)	0.141	2.35	(1.08–5.14)	0.032
**High-frequency hearing loss**						
0<SI≤150 (Ref.)	1			1		
SI>150	1.89	(1.23–2.91)	0.004	0.85	(0.25–2.97)	0.802

Smoking Index (SI): cigarettes/day multiplied by number of smoking years. AOR: adjusted odds ratio; adjusted for the covariates of age, education level, income level, drinking, workplace noise, frequency of earphone use, hypertension, diabetes, and hyperlipidemia.

* Statistical significance at p<0.05. Ref.: reference.

## DISCUSSION

We investigated the association between cigarette smoking and hearing loss in the Chinese general population. Smoking status varied according to age and gender. Cigarette smoking was significantly associated with both speech-frequency and high-frequency hearing loss in males, but not in females. In middle-aged males, cigarette smoking was significantly associated with both speech-frequency and high-frequency hearing loss. Past smokers showed the highest risk for hearing loss. In older males, smoking status was significantly associated with speech-frequency hearing loss, but not with high-frequency hearing loss. Passive smokers showed no significant risk of hearing loss compared with never smokers. Current smoking had a dose-dependent relationship with speech-frequency hearing loss in older males, and high-frequency hearing loss in middle-aged males.

We found that cigarette smoking was significantly associated with both speech-frequency and high-frequency hearing loss only in males. This is consistent with the results obtained by Wang et al.^8^ who also found the association was not significant in females in China. In the study by Wang et al.^8^, the smoking rate in females was 2.6%, and in our study the smoking rate was 2.0%. We speculated that this discrepancy may be the result of the substantially lower smoking rate in females in our sample, which resulted in limited statistical power. A larger sample size of female smokers should be investigated in the future to clarify the relationship between smoking and hearing loss in females.

Current smoking was found to be associated with an increased prevalence of hearing loss in middle-aged and older males compared to never smokers, and this relationship is supported by previous cross-sectional studies and cohort studies^5,9,10,15,16^. Chang et al.^10^ investigated hearing loss among 12935 participants in the Korea National Health and Nutrition Examination Survey (KNHANES) and found that current smoking is associated with speech-frequency and high-frequency hearing impairment across all ages. Moreover, Hu et al.^5^ conducted a cohort study including 50195 employees aged 20– 64 years who were followed up for a maximum of 8 years, and revealed that smoking is associated with an increased risk of hearing loss at 1kHz and 4kHz.

The relationship between cigarette smoking and hearing loss varied according to age in males, specifically in middle-aged and older males, but not in young males. The risk of hearing loss increased with age^17^, and young males had the lowest prevalence of hearing loss. The damage done by tobacco to human health does not become evident until years or even decades after the onset of its use^18^. In young males, drinking, workplace noise, hypertension, and diabetes, were significantly associated with hearing loss, but smoking was not. We believe that the negative effect of smoking on hearing loss is not apparent in young adults. In the middle-aged group, the negative effect of smoking has accumulated for years, and the effect on hearing loss became evident together with other risk factors like workplace noise, hypertension, and diabetes. In older males, only past smoking showed a significantly higher risk for speech-frequency hearing loss, but not current smoking. We speculated that the negative effect of cigarette smoking on hearing loss was likely overwhelmed by the dominant agingrelated hearing loss which results from physiological dysfunction or chronic diseases like hypertension and hyperlipidemia. Similarly, Wang et al.^8^ found that the association of current smoking with speech-frequency hearing loss was significant in individuals younger than 70 years but not in those aged ≥70 years.

Additionally, past smokers had the highest risk of hearing loss compared with never smokers, but not current smokers. This is similar to the results obtained by Shargorodsky et al.^5^ in their cohort study. It was found that a history of past smoking was statistically significantly associated with an increased risk of self-reported hearing loss in adult males, but not in current smokers. In the present study, most past smokers in middle-aged or older male subgroup likely had higher average age and longer duration of cigarette exposure prior to quitting smoking, and the hazardous components of cigarettes have already affected the cochlear circulation, thereby resulting in a higher prevalence of hearing loss. This inference is also supported by the fact that in the young age group, past smokers did not have a significantly higher risk of hearing loss compared with never smokers. This finding could be explained by timely smoking cessation, and the duration of cigarette exposure was likely to have been too short to have a negative effect on hearing loss. Therefore, quitting smoking as young as possible is essential for hearing health maintenance. In contrast, Hu et al.^5^ found that past smokers had lower risk of hearing loss than current smokers, and the excess risk of hearing loss associated with smoking disappears in a relatively short period of time after quitting. This discrepancy needs further explanation.

In this study, compared with never smokers, passive smokers did not have a statistically significantly higher risk of speech-frequency and high-frequency hearing loss. This result is inconsistent with the findings of Dawes et al.^11^ who found that non-smokers who reported passive exposure to tobacco smoke were more likely to have higher risk of hearing loss. Chang et al.^10^ found that passive smokers have an elevated prevalence of either speech-frequency bilateral hearing loss or high frequency hearing loss only in people in their 40s. The passive smoking rate in China is at least 50% in the never smokers^7^, however, in our study, the average passive smoking rate is <30%. We speculated that some never smokers in our study likely neglected to report their passive smoking exposure in the questionnaire, which led to the lack of a detectable significant difference.

Regarding smoking intensity, current smoking was positively associated with an increased risk of both types of hearing loss in middle-aged and older males in a dose-dependent manner. This is consistent with previous studies that reported the risk for hearing loss increased with the number of cigarettes smoked per day^5,7^ and pack-years^11^. Mizoue et al.^19^ found that the risk for high-frequency hearing loss, but not low-frequency hearing loss, is elevated with increasing numbers of cigarettes smoked per day. Hu et al.^5^ found that smoking was associated with an increased risk of hearing loss in a dose-dependent manner and that the association is more pronounced for high-frequency hearing loss than for low-frequency hearing loss.

The association between smoking and high-frequency hearing loss was stronger than low-frequency hearing loss. This is likely because cigarette smoking has a direct ototoxic effect on outer hair cell function^20^, or damages the auditory system by increasing carboxyhemoglobin and reducing blood flow to cochlea^21,22^, which negatively affects high-frequency hearing loss.

### Strengths and limitations

This study has several strengths. First, it was a large-scale, general population-based survey conducted in China that fills the gap left by the severe shortage of studies on the relationship between cigarette smoking and hearing loss, especially in different gender and age groups. We analyzed the relationship between cigarette smoking and hearing loss in stratified age subgroups rather than adjusting for the age, resulting in a more detailed description of the effect of smoking on hearing loss. Second, we selected pure-tone threshold audiometry at nine frequency bands instead of self-reported hearing loss or narrow frequency bands, which enabled a more precise evaluation of hearing loss. Finally, the questionnaire used included an extensive set of covariates which excluded many potential confounders.

Nevertheless, this study also has some limitations. First, the cross-sectional design has limited the evidence for causal inference. Second, a questionnaire was used to collect information on smoking habits and history of diseases based on self-reporting by the participants, without secondary sources to confirm the accuracy of responses. Third, more than 10 variables were included in this study, and the regression analysis was performed under the hypothesis that all these included covariates were independent of each other. Finally, for past smokers, we did not collect the smoking duration before quitting. Therefore, we were unable to determine why they had the highest prevalence of hearing loss.

## CONCLUSIONS

The strongest association between cigarette smoking and speech-frequency and high-frequency hearing loss was observed in middle-aged males in a dose-dependent manner, the past smokers showed the highest prevalence. Different smoking intervention measures should be taken for different age groups.

## References

[1] World Health Organization (2019). New WHO-ITU standard aims to prevent hearing loss among 1.1 billion young people. In Chinese.

[2] Nash SD, Cruickshanks KJ, Huang GH (2013). Unmet hearing health care needs: the beaver dam offspring study. Am J Public Health.

[3] Moon IJ, Baek SY, Cho YS (2015). Hearing aid use and associated factors in South Korea. Medicine (Baltimore).

[4] World Health Organization (2013). Millions of people in the world have hearing loss that can be treated or prevented.

[5] Hu H, Sasaki N, Ogasawara T (2019). Smoking, Smoking Cessation, and the Risk of Hearing Loss: Japan Epidemiology Collaboration on Occupational Health Study. Nicotine Tob Res.

[6] GBD 2016 Disease and Injury Incidence and Prevalence Collaborators (2017). Global, regional, and national incidence, prevalence, and years lived with disability for 328 diseases and injuries for 195 countries, 1990-2016: a systematic analysis for the Global Burden of Disease Study 2016. Lancet.

[7] Yang G, Wang Y, Wu Y, Yang J, Wan X (2015). The road to effective tobacco control in china. Lancet.

[8] Wang D, Wang Z, Zhou M (2017). The combined effect of cigarette smoking and occupational noise exposure on hearing loss: evidence from the Dongfeng-Tongji Cohort Study. Sci Rep.

[9] Sumit AF, Das A, Sharmin Z (2015). Cigarette smoking causes hearing impairment among Bangladeshi population. Plos One.

[10] Chang J, Ryou N, Jun HJ, Hwang SY, Song JJ, Chae SW (2016). Effect of Cigarette Smoking and Passive Smoking on Hearing Impairment: Data from a Population Based Study. Plos One.

[11] Dawes P, Cruickshanks KJ, Moore DR (2014). Cigarette smoking, passive smoking, alcohol consumption, and hearing loss. J Assoc Res Otolaryngol.

[12] Gopinath B, Flood VM, Mcmahon CM, Burlutsky G, Smith W, Mitchell P (2010). The effects of smoking and alcohol consumption on age-related hearing loss: the Blue Mountains Hearing Study. Ear Hear.

[13] Shargorodsky J, Curhan SG, Eavey R, Curhan GC (2010). A prospective study of cardiovascular risk factors and incident hearing loss in men. Laryngoscope.

[14] GBD 2015 Tobacco Collaborators (2017). Smoking prevalence and attributable disease burden in 195 countries and territories, 1990-2015: a systematic analysis from the Global Burden of Disease Study 2015. Lancet.

[15] Nomura K, Nakao M, Morimoto T (2005). Effect of smoking on hearing loss: quality assessment and meta-analysis. Prev Med.

[16] Burr H, Lund SP, Sperling BB, Kristensen TS, Poulsen OM (2005). Smoking and height as risk factors for prevalence and 5-year incidence of hearing loss. A questionnaire-based follow-up study of employees in Denmark aged 18-59 years exposed and unexposed to noise. Int J Audiol.

[17] Hong JW, Jeon JH, Ku CR, Noh JH, Yoo HJ, Kim DJ (2015). The prevalence and factors associated with hearing impairment in the Korean adults: the 2010-2012 Korea National Health and Nutrition Examination Survey (observational study). Medicine (Baltimore).

[18] World Health Organization (2008). WHO Report on the global tobacco epidemic, 2008: the MPOWER package.

[19] Mizoue T, Miyamoto T, Shimizu T (2003). Combined effect of smoking and occupational exposure to noise on hearing loss in steel factory workers. Occup Environ Med.

[20] Lustig LR (2006). Nicotinic acetylcholine receptor structure and function in the efferent auditory system. Anat Rec A Discov Mol Cell Evol Biol.

[21] Fechter LD, Thorne PR, Nuttall AL (1987). Effects of carbon monoxide on cochlear electrophysiology and blood flow. Hear Res.

[22] Palmer KT, Griffin MJ, Syddall HE, Coggon D (2004). Cigarette smoking, occupational exposure to noise, and self reported hearing difficulties. Occup Environ Med.

